# AI-Driven Innovation in Skin Kinetics for Transdermal Drug Delivery: Overcoming Barriers and Enhancing Precision

**DOI:** 10.3390/pharmaceutics17020188

**Published:** 2025-02-02

**Authors:** Nubul Albayati, Sesha Rajeswari Talluri, Nirali Dholaria, Bozena Michniak-Kohn

**Affiliations:** 1 Ernest Mario School of Pharmacy, Rutgers-The State University of New Jersey, 160 Frelinghuysen Road, Piscataway, NJ 08854, USA; na501@gsbs.rutgers.edu (N.A.); sesha.rajeswaritalluri@rutgers.edu (S.R.T.); nvd16@scarletmail.rutgers.edu (N.D.); 2 Center for Dermal Research, Rutgers-The State University of New Jersey, 145 Bevier Road, Piscataway, NJ 08854, USA

**Keywords:** artificial intelligence, transdermal drug delivery, machine learning, formula optimization, BioSIM

## Abstract

Transdermal drug delivery systems (TDDS) offer an alternative to conventional oral and injectable drug administration by bypassing the gastrointestinal tract and liver metabolism, improving bioavailability, and minimizing systemic side effects. However, widespread adoption of TDDS is limited by challenges such as the skin’s permeability barrier, particularly the stratum corneum, and the need for optimized formulations. Factors like skin type, hydration levels, and age further complicate the development of universally effective solutions. Advances in artificial intelligence (AI) address these challenges through predictive modeling and personalized medicine approaches. Machine learning models trained on extensive molecular datasets predict skin permeability and accelerate the selection of suitable drug candidates. AI-driven algorithms optimize formulations, including penetration enhancers and advanced delivery technologies like microneedles and liposomes, while ensuring safety and efficacy. Personalized TDDS design tailors drug delivery to individual patient profiles, enhancing therapeutic precision. Innovative systems, such as sensor-integrated patches, dynamically adjust drug release based on real-time feedback, ensuring optimal outcomes. AI also streamlines the pharmaceutical process, from disease diagnosis to the prediction of drug distribution in skin layers, enabling efficient formulation development. This review highlights AI’s transformative role in TDDS, including applications of models such as Deep Neural Networks (DNN), Artificial Neural Networks (ANN), BioSIM, COMSOL, K-Nearest Neighbors (KNN), and Set Covering Machine (SVM). These technologies revolutionize TDDS for both skin and non-skin diseases, demonstrating AI’s potential to overcome existing barriers and improve patient care through innovative drug delivery solutions.

## 1. Introduction

By enabling drugs to permeate through the skin and enter systemic circulation, TDDS provides significant advantages over traditional oral and injectable routes [1]. One key benefit is bypassing the first-pass metabolism in the liver, which can degrade active drug compounds, leading to reduced bioavailability [2]. Additionally, TDDS supports controlled and sustained drug release, maintaining therapeutic drug levels over an extended period and improving patient compliance by reducing the frequency of administration [3,4,5,6,7,8].

Despite these advantages, the efficacy of TDDS is limited by several critical factors. The most formidable challenge is the stratum corneum, the outermost layer of the skin, which acts as a robust barrier to most drugs [9]. Its low permeability restricts the passage of large and hydrophilic molecules, necessitating the use of penetration enhancers, advanced delivery systems, or chemical modifications of the drug [10,11,12]. Furthermore, developing effective TDDS formulations is complex, requiring a delicate balance between drug stability, release rates, and compatibility with skin physiology.

Over the past decade, research and technological advancements in transdermal drug delivery systems (TDDS) have significantly expanded. This area has seen the publication of numerous research articles and the development of innovative technologies, reflecting a strong global interest in overcoming the challenges associated with TDDS. Studies indicate a substantial increase in TDDS-related research over the past ten years, emphasizing limitations such as skin permeability and the delivery of macromolecular drugs. Techniques such as microneedles, chemical penetration enhancers (CPEs), and nanotechnology have garnered considerable research attention. Microneedles, for example, have been extensively studied for applications ranging from vaccine delivery to pain management, making them a central focus of TDDS research [13,14,15,16]. Over the past decade, research in TDDS, particularly for vaccine delivery using microneedles, has significantly increased [17]. Studies highlight advances in materials, fabrication techniques, and integration with biologics, reflecting TDDS’s growing role in non-invasive, patient-friendly vaccine administration [18]. Emerging technologies in transdermal drug delivery systems (TDDS) are transforming the field with innovative approaches [19]. Nanocarriers such as liposomes and nanoparticles improve drug stability and targeted delivery for applications in cancer and dermatology. Techniques like electroporation and photomechanical waves temporarily disrupt the skin barrier for molecule delivery, while thermal ablation employs localized heat to enhance penetration, enabling rapid and localized drug administration [20,21]. Integrating these novel approaches with AI and machine learning is also emerging as a transformative trend, facilitating formulation optimization, real-time adjustments, and enhanced patient-specific solutions. The growing body of TDDS research and innovative technology development highlights its potential to revolutionize non-invasive drug delivery.

In developing a modern TDDS for effective disease treatment, employing a predictive computational model that accurately simulates material interactions and the drug’s skin permeability and release profile over time can save time and resources [22]. Over the last decade, drug delivery has increasingly leveraged computational technology to create innovative and targeted delivery systems [23].

As we progress into a new century marked by remarkable technological advancements, Machine Learning (ML) and Artificial Intelligence (AI) have emerged as powerful tools for managing and analyzing scientific data. The introduction of AI and ML methodologies has revolutionized various sectors within the medical field, mainly benefiting the pharmaceutical industry. The term “Artificial Intelligence” was first coined by John McCarthy in 1956 [24,25,26]. AI is the capacity of the computer to mimic human cognitive skills and thought processes and to translate into informative solutions from complex sets of data [27].

**Figure 1:**
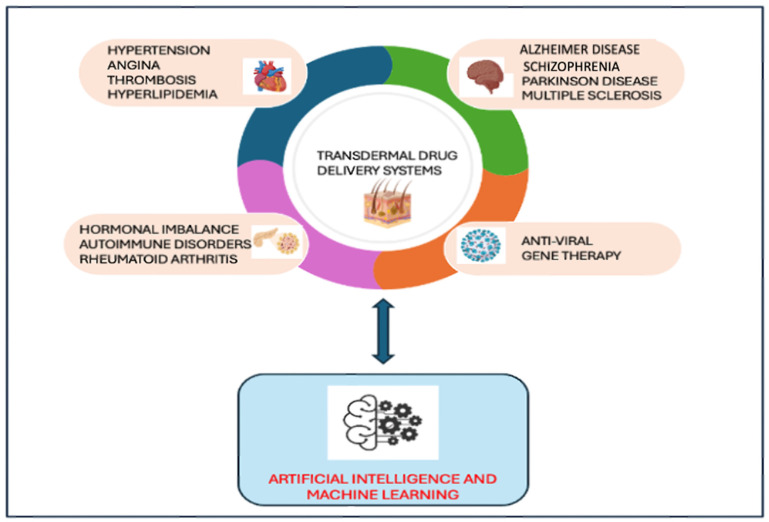
Integration of AI and MLTDDS-based based therapies.

AI is a computer system replicating human cognitive functions and thought processes, transforming complex data sets into meaningful insights [27]. Artificial intelligence (AI) and machine learning (ML) advancements have begun changing the landscape of TDDS research and development. These technologies streamline the design and optimization processes by analyzing large datasets, predicting drug-skin interactions, and identifying ideal formulation components. AI algorithms can model the permeation behavior of drugs, assess the impact of excipients, and optimize the physical and chemical properties of delivery systems. With the rising interest in AI’s capabilities to analyze and interpret a wide range of information—from genetic data to clinical applications of therapeutics—AI can significantly accelerate the traditionally lengthy drug discovery-to-marketing process Figure 1 [28,29].

AI can significantly transform the process of determining drug suitability for skin delivery formulation, impacting everything from drug manufacturing to marketing. As a powerful tool, AI presents a remarkable opportunity for advancements in drug discovery, redesigning, formulation, and testing of pharmaceutical dosage forms [30].

With deep neural network (DNN) models, AI can detect skin diseases and predict the condition of the skin based on digital images. This AI-assisted information plays a crucial role in making informed decisions at the early stages of drug discovery and formulation and in modulating a drug’s therapeutic exposure within the body. AI is particularly valuable in formulation design and optimization, focusing on smart drug-carrier choices for dermal delivery, which has become a trend in addressing various dermal diseases and enhancing the skin-care cosmetic industry. The accurate prediction of drug deposition in the skin from various transdermal drug delivery systems (TDDS) is essential information for formulation development. AI and machine learning (ML) algorithms now provide the means to unlock this vital information, offering actionable insights for effective disease detection and formulation design [22].

Dynamic AI and ML-derived models are discussed in this review, for example, the pharmacokinetic prediction model BIOiSIM for drug exposure & distribution prediction [31], a computation model COMSOL providing estimates about the temperature effect on the skin-absorption of the drug [32], utilization of a computation model referenced as ANN (Artificial Neural Network) for predicting the efficacy of two medications for COVID-19 [33], have fueled the importance of such AI and ML based approaches in a pharmaceutical field. Other ML-based models such as K-Nearest Neighbors (KNN), Set Covering Machine (SVM), and Deep Neural Network (DNN) are discussed for their ability to predict the active protease inhibitor site for the treatment of HIV-1 [34].

Furthermore, AI-driven systems enable the development of smart transdermal devices that monitor real-time physiological parameters and adjust drug release dynamically to ensure consistent therapeutic outcomes. This convergence of AI and TDDS holds the potential to overcome traditional barriers and pave the way for innovative, personalized, and efficient drug delivery solutions.

## 2. Emerging TDDS for the Treatment of Viral Infections and Other Vital Organ Disorders

### 2.1. TDDS for the Treatment of Viral Infections

TDDS are particularly effective for treating skin-based viral infections, as it allows for targeted application directly to affected areas. This method also minimizes systemic absorption and reduces related side effects [35]. By utilizing advanced techniques to deliver innovative nanocarriers to the target site, the goal is to significantly lower viral loads more effectively than standard drug dosage forms [36,37,38]. Transdermal administration is considered the most suitable for controlling cutaneous viral infections such as herpes, chickenpox [37,39], warts [38,40], and influenza [41]. Recent studies suggest that transdermal delivery could be an alternative route for administering COVID-19 vaccines [37,39,41,42]. A penciclovir-loaded hydrogel nanoemulsion (HN) was developed for transdermal herpes simplex treatment, demonstrating double the permeation efficiency of a marketed cream in vitro using porcine skin. This promising formulation demonstrated enhanced drug delivery through comparative percutaneous absorption studies [43]. A comparative study examined binary ethosomes (ETHOs) and elastic liposomes (ELPs) for transdermal delivery of sodium salt Acyclovir (ACV-Na). The evaluation included in-vitro release, ex-vivo permeation, and skin deposition, showing that optimized ETHOs significantly enhanced permeation flux and drug deposition compared to ELPs. Safety assessments through hemolysis and skin irritation analyses indicated that ETHOs are a promising alternative to traditional vesicular systems for improved skin permeation and stability in transdermal applications. A study formulated an oil-in-water (o/w) microemulsion using these enhancers for transdermal delivery of acyclovir, which was tested for efficacy in a murine model of zosteriform cutaneous HSV-1 infection [41,44]. Influenza vaccine-loaded egg microneedles (FLU-EMN) were designed and characterized, highlighting their potential for effective and minimally invasive delivery of therapeutic drugs without the need for cold storage [45]. This microneedle system could become an effective method for nationwide vaccination, making the process more cost-effective, safe, and feasible. Microneedles rapidly activate therapeutic properties. A minimally invasive microneedle array patch (MAP) was developed to deliver Zanamivir, achieving higher lung tissue concentrations and demonstrating potential as an innovative treatment for seasonal influenza [46]. Microneedle drug delivery has shown promise in treating plantar warts caused by HPV. Ghonemy et al. reported that combining microneedling with 5-fluorouracil (5-FU) achieved an 86.7% complete response rate, outperforming intralesional 5-FU (76.7%) and microneedling alone (70%), with improved patient satisfaction due to reduced discomfort [40]. Numerous SARS-CoV-2 vaccines have been developed since the COVID-19 outbreak, with 242 candidates in clinical development and 11 approved for emergency use by WHO. While most vaccines require subcutaneous or intramuscular administration, transdermal delivery offers a promising alternative, particularly for self-administration in resource-limited areas. A fluorocarbon-modified chitosan-based transdermal system was shown to effectively deliver the SARS-CoV-2 vaccine, generating immune responses comparable to subcutaneous injections by activating immune cells and targeting lymph nodes [47]. A microneedle array (MNA) was developed to deliver the SARS-CoV-2 S1 subunit vaccine, targeting the spike protein to prevent infection. MNAs expedite vaccine production and reduce costs by requiring fewer doses. These MNA-based vaccines generate strong antibody responses, and stability studies suggest that integrating vaccine components into the MNA polymer matrix preserves their structure and binding functions. These MNAs are designed for self-administration, eliminating the need for specialized equipment [42]. A comprehensive list of viral infections treated using TDDS is presented in Table 1 below.

### 2.2. TDDS for the Treatment of Central Nervous System (CNS) Disorders

Significant Progress has been made in developing transdermal formulations for treating central nervous system disorders. Transdermal drug delivery systems (TDDS) that include nicotinic agonists, particularly nicotine, are commonly used to assist in smoking cessation and to help manage withdrawal symptoms [49,50]. These agents have also shown potential in alleviating cognitive impairments associated with neurodegenerative diseases and psychiatric conditions such as schizophrenia and attention disorders [51,52,53]. Transdermal nicotine has been observed to improve symptoms of ADHD, although the underlying mechanisms remain unclear. This improvement may be linked to nicotine’s ability to increase dopamine release, which is also enhanced by conventional ADHD treatments like methylphenidate and amphetamine [53]. Research conducted by eminent scientists has introduced a new methylphenidate patch designed for long-term Attention-Deficit/Hyperactivity Disorder (ADHD) management. This patch functions as both an adhesive and a drug reservoir, allowing dosage adjustments based on patch size and wear duration to personalize treatment [51].

Furthermore, a study led by Sittl et al. demonstrated the effectiveness of a transdermal buprenorphine system for managing severe chronic pain from conditions like cancer, reducing the need for oral medications. This system has been associated with improved sleep duration and generally mild to moderate side effects commonly seen with opioid therapy [49]. Another notable development is a transdermal system integrating the antidepressant selegiline, which effectively inhibits central monoamine oxidase A (MAO-A) and B (MAO-B) enzymes. While its antidepressant effects are modest, it avoids metabolism by the liver and intestines, enhancing its therapeutic advantage [54]. Several commercially available TDDSs target the central nervous system (CNS), such as Neupro, a rotigotine system for early-stage Parkinson’s disease, and nicotine patches (Nicotinell, Nicodern, Prostep) for smoking cessation. Other products include Matrifen and Duragesic systems containing fentanyl for pain management and Transderm-Scop for motion sickness. Specialized transdermal buprenorphine devices are also available for treating sickle cell disease-related pain [55]. Early clinical trials have shown limited success for acetylcholinesterase inhibitors (AChEIs) like TTS-physostigmine and TTS-tacrine, lacking sufficient evidence of efficacy beyond a placebo [56,57]. However, transdermal rivastigmine has been effective in reducing cognitive and functional impairments in Alzheimer’s disease patients [58]. A comprehensive list of drugs administered through TDDS is detailed in Table 2.

### 2.3. TDDS for the Treatment of Cardiovascular (CV) Diseases

In a heart failure (HF) scenario, the drug’s pharmacokinetics (PK), pharmacodynamics (PD), and tolerability are frequently impacted by changes in the body’s physiological parameters from hypoperfusion systemic conditions due to reduced cardiac ejection fraction [77]. In addition, drug metabolism and metabolite clearance are reduced in renal failure [78]—co-morbid conditions like hypoalbuminemia and hepatic congestion impair drug absorption [79].

Therefore, transdermal patch delivery systems may offer an effective solution for drug administration. Propranolol, a nonselective beta-adrenergic blocker, undergoes significant hepatic first-pass metabolism when taken orally, leading to a bioavailability of about 23% [80]. An animal study with rabbits demonstrated that oral propranolol achieved a Cmax of 56.4 ng/mL within 13.2 min, but due to liver metabolism, its bioavailability was only 12.3% [81]. Conversely, the transdermal propranolol patch reached a steady-state plasma concentration (Css) of 9.3 ng/mL after an initial lag time of 8 h, with a bioavailability that was 74.8% higher than that of oral propranolol [82].

Nitroglycerin is another significant drug in cardiovascular therapy. Lauder & Blanton used nitroglycerin to relieve angina pectoris and first observed drug resistance after repeated doses in 1867 [83]. Ferid Murad discovered that nitric oxide (NO) from nitroglycerin acts on vascular smooth muscle by activating cyclic guanosine monophosphate (cGMP), leading to vasodilation [84]. A two-way crossover study was conducted on twenty-five healthy males using Nitro-Dur and another type of nitroglycerin transdermal patch, Nitro-Dur II, which showed average Cmax values of 0.383 ng/mL and 0.432 ng/mL, respectively [85].

Bisono^®^ Tape is a transdermal patch containing bisoprolol as an active ingredient [86], used to manage conditions such as aortic dissection [87], premature ventricular contraction [88], orthostatic hypotension due to heart failure [89], and atrial fibrillation [90]. A comparative study involving edematous and non-edematous patients using the Bisono^®^ Tape 4 mg patch showed that the Cmax was 13.3 ng/mL in the edema group and 17 ng/mL in the non-edema group [91]. This study aimed to investigate the effect of systemic edema on the absorption of beta-blockers from skin patches in critically ill patients. However, it was found that systemic edema of the lower extremities did not affect the blood levels or the heart rate-lowering effects of bisoprolol after applying the bisoprolol skin patch.

Another antihypertensive drug that utilizes transdermal patch delivery is clonidine. Clonidine is an α2-adrenergic agonist initially used to treat hypertension [91]. It has also been used for conditions such as attention-deficit hyperactivity disorder (ADHD) [92] and drug withdrawal syndrome [93]. The transdermal clonidine patch was introduced in 1983 and approved by the FDA in 1984 [94,95]. Since then, a comparative study of oral and transdermal clonidine has been conducted [96]. The results showed no difference in Cmax between oral clonidine (0.39 ng/mL) and transdermal clonidine (0.3 ng/mL), but the half-life of transdermal clonidine was longer than that of oral clonidine (31.9 h vs. 10.8 h). Additionally, there was no difference in the antihypertensive effect [96].

Losartan, an angiotensin II receptor blocker (ARB), is also being developed for transdermal drug delivery. A previous study on rat skin with proniosome transdermal drug delivery showed that transdermal losartan achieved a Cmax of 141 ng/mL compared to 152 ng/mL when taken orally. However, the bioavailability of transdermal losartan was 1.93 times that of oral losartan [97]. Various cardiovascular drugs formulated as transdermal delivery systems are listed in Table 3.

### 2.4. TDDS for the Treatment of Other Complications Such as Hormonal Imbalance and Autoimmune Disorders

Transdermal drug delivery systems (TDDS) have shown promise in managing conditions like hormonal imbalances and autoimmune disorders. For hormonal imbalances, TDDS offers an effective method for administering hormones such as estrogen, testosterone, and progesterone, often in patches or gels [109]. These systems allow for controlled, continuous release of the hormone, bypassing first-pass metabolism in the liver and providing a steady, more predictable therapeutic effect. For example, transdermal patches for hormone replacement therapy (HRT) are widely used for menopausal symptoms and androgen deficiency in men [110]. In autoimmune disorders like rheumatoid arthritis and psoriasis, TDDS has been explored to deliver anti-inflammatory and immunosuppressive drugs directly to the affected areas, minimizing systemic side effects [111]. Recent innovations, such as microneedles and smart patches, enhance the precision of drug delivery, improve patient compliance, and allow personalized treatments [112]. The potential for integrating AI-driven formulation optimization further enhances the effectiveness of TDDS in managing these chronic and complex conditions. Table 4 is focused on TDDS for the treatment of hormonal imbalance and pain management with examples of drugs and transdermal drug delivery systems (TDDS), along with references:

## 3. Application of AI in Various Stages of TDDS

### 3.1. Screening of Drug Molecules and Optimizing Formula

AI can identify molecules with ideal physicochemical properties (e.g., solubility and lipophilicity) for transdermal delivery, saving time and resources in drug development. Machine learning (ML) algorithms predict the optimal formulation of drugs and excipients, enhancing drug permeation through the skin [30]. Techniques like quantitative structure-activity relationships (QSAR) modeling and advanced deep learning methods allow researchers to predict molecular interactions and properties [125], streamlining the identification of suitable candidates for TDDS. Machine learning (ML) algorithms can predict the optimal formulation of drugs and excipients, enhancing drug permeation through the skin.

### 3.2. Skin Permeability Prediction

AI models analyze large datasets of skin permeability for various compounds, predicting their suitability for transdermal delivery. AI helps adjust formulations based on the skin type, condition (e.g., hydration), and target drug. The study conducted by Abdallah et al. focuses on using AI algorithms to predict skin permeability (LogKp) for FDA-approved drugs based on molecular structures. It applies regression models and clustering techniques to analyze permeability patterns, aiding in selecting drug candidates for transdermal formulations [126]. A study conducted by Defraeye et al. focuses on using AI algorithms to predict skin permeability (LogKp) for FDA-approved drugs based on molecular structures [127]. A recent review of developments in modeling the skin’s microstructure using finite element modeling emphasized integrating advanced techniques to simulate the absorption of drugs and cosmetics, improving the representation of the skin’s physical properties [128]. A comprehensive dataset on FDA-approved drugs was analyzed using advanced machine learning techniques such as ensemble methods (e.g., Random Forest and XGBoost) and artificial neural networks (ANNs). These models predicted skin permeability (LogKp values) and demonstrated enhanced accuracy for drug diffusion properties in transdermal systems [126].

### 3.3. Design and Optimization of Delivery Systems

AI significantly enhances the design and optimization of transdermal patches by analyzing multiple parameters such as geometry, adhesive properties, and material composition [129]. AI-driven models predict the impact of patch design variables on drug release profiles, ensuring consistent delivery rates. For instance, machine learning algorithms can identify the optimal combination of adhesive layers and matrix materials to balance adhesion with drug diffusion. Moreover, AI enables the customization of patches for specific patient needs, such as varying skin types and drug absorption rates. Advanced simulation techniques also allow researchers to predict patch performance under different environmental and physiological conditions [130]. The design of microneedles benefits from machine learning, which refines parameters such as length, tip sharpness, and material selection to maximize efficiency while minimizing discomfort. AI tools assess the interaction between microneedles and skin to optimize penetration depth and drug diffusion without causing significant pain or damage. Moreover, AI facilitates the development of biodegradable microneedles, ensuring safe and effective drug delivery. Simulation-based approaches allow rapid prototyping of microneedle arrays, reducing development time and resource usage. These innovations make microneedles suitable for applications ranging from vaccine delivery to hormone therapies [131,132,133,134].

### 3.4. Personalized Medicine

Smart patches integrated with AI-enabled sensors can monitor drug levels in the body and adjust delivery dynamically. Smart patches with AI-enabled sensors represent a revolutionary leap in TDDS [135]. These systems dynamically monitor drug levels in real-time and adapt delivery rates as needed, ensuring that therapeutic levels are maintained consistently. This feedback mechanism minimizes risks of over- or under-dosing while improving patient outcomes. The integration of such technologies not only enhances treatment efficacy but also improves patient adherence by reducing complexity. AI systems analyze patient data (e.g., skin type, age, and medical history) to tailor drug doses and delivery schedules. AI algorithms leverage patient-specific data, including skin characteristics, genetic profiles, and medical history, to design individualized drug regimens. These systems could be used to analyze variations in skin permeability and health conditions, enabling precise optimization of drug dosage and delivery schedules tailored to each patient’s unique needs [136].

### 3.5. Simulation and Virtual Testing

Skin Models: There are multiple test models for predicting dermal absorption, many of which depend on ex vivo animal or human models. However, growing restrictions on animal and human skin use, combined with significant differences in dermal composition—such as hair follicle density and skin thickness between various species—complicate accurate predictions of pharmacokinetics (PK) in humans. In recent years, the incorporation of AI algorithms has transformed the pharmaceutical industry, reinventing drug development through large-scale systems and a big data-driven approach. AI studies how computers and machine learning systems imitate human intelligence using computational techniques to solve human problems. Hence, using AI and machine learning, one can explore predicting dermal exposure of therapeutics and evaluating the impact of different formulations on transdermal disposition.

AI-driven simulations replicate drug interactions with skin layers, including the stratum corneum, epidermis, and dermis. These models provide detailed insights into the mechanisms of drug permeation, reducing dependency on traditional in vitro or in vivo experiments. For instance, computational techniques such as compartmental and diffusion models use differential equations to describe the movement of drugs through skin layers [137]. These simulations effectively assess the penetration of various drug types and evaluate their potential for transdermal delivery, often replacing or complementing “skin-on-a-chip” technologies [138]. AI-powered predictive models forecast outcomes of drug delivery in diverse scenarios, such as varying patient conditions and drug formulations. Such models utilize large datasets to train machine learning algorithms that evaluate parameters like drug permeability, stability, and absorption. By predicting the performance of formulations before physical trials, these methods streamline the development process and minimize costs. Recent innovations, such as microfluidic “skin-on-a-chip” systems, mimic the physiological properties of human skin more accurately than traditional methods [139]. These devices integrate sensors to monitor real-time changes and effects of drug delivery, providing high-resolution data for validating AI-based predictions. These advancements underscore the potential of combining AI with advanced simulation techniques to revolutionize TDDS, making drug development faster, more cost-effective, and ethically sustainable. In a study by Maharao et al. [140], a revolutionary transdermal model was developed and validated using a BIOiSIM platform. This platform operates on dynamic drug pharmacokinetic predictions driven by biological inputs. Integrating machine learning and physiological modeling, BIOiSIM offers a scalable computational solution to make accurate and rapid predictions. It employs a 16-compartment model to simulate and predict the exposure of three distinct drug-like compounds—morphine, buprenorphine, and nicotine—both chemically and biologically. The model effectively predicts their distribution with an average fold error of less than two, showcasing its accuracy in forecasting compound distributions through the skin [140].

### 3.6. Efficiency in Clinical Trials

AI has significantly enhanced the efficiency of clinical trials in various aspects, especially for transdermal drug delivery systems (TDDS). Regarding patient recruitment, AI excels by analyzing vast datasets, including electronic health records (EHRs), social media platforms, and genetic data, to identify suitable candidates faster and more accurately. This targeted approach improves trial validity by ensuring that participants are more likely to benefit from the tested treatment [141]. Moreover, AI can enhance retention by detecting at-risk patients early and providing timely interventions, such as automated reminders, thus improving adherence to the trial protocol. AI also streamlines data collection and analysis, minimizing errors and significantly reducing the time required for these processes. By automating data integration from various sources like medical records and wearables, AI ensures consistency and accuracy, which is crucial for maintaining high-quality data. Additionally, AI aids in predictive modeling, helping researchers anticipate trial outcomes and adapt designs based on interim results, thus preventing delays or protocol failures. These AI-driven advancements have the potential to shorten trial durations, reduce costs, and improve overall trial success rates, especially by optimizing recruitment, data analysis, and outcome prediction [142].

### 3.7. Approaches to Resolve Problems

AI significantly enhances drug penetration and reduces variability in transdermal drug delivery systems (TDDS). Penetration enhancers are critical for improving the skin’s permeability to drugs, especially those with low natural penetration properties. AI aids in identifying new enhancers by analyzing molecular interactions and predicting their effectiveness. For instance, AI-based models can predict how certain compounds, like dimethyl sulfoxide (DMSO) or azone, influence the structure of the stratum corneum and promote better drug transport across the skin. Additionally, AI helps address variability in skin response, which varies across populations due to age, skin type, and environmental conditions. Machine learning algorithms can analyze large datasets of skin responses to different transdermal systems, identifying patterns that lead to more consistent and effective drug delivery across diverse patient groups. These technologies enable the development of more personalized and efficient TDDS tailored to specific patient needs and conditions [126].

Over the decades, various mechanisms have been proposed to explain how temperature influences skin permeability and flux in ex vivo human skin. However, there is still limited understanding of why temperature influences drug absorption. However, understanding the specific reasons behind temperature affecting drug absorption remains limited. Therefore, connecting the transient and steady-state temperature changes to drug transport across the stratum corneum (SC) and its effects on dermal clearance and systemic pharmacokinetics (PK) is crucial. La Count et al. introduced a model to address simultaneous heat and mass transport using COMSOL software version 5.3, focusing on the effects of transient and steady-state temperatures on nicotine absorption in the skin. Their model, grounded in extensive literature analysis, was validated against human in vivo and in vitro data for nicotine. Notably, the results indicated that a 10 °C increase in skin surface temperature correlates with a twofold increase in nicotine absorption, suggesting an activation energy of 50–65 kJ/mol for diffusion in the stratum corneum, which acts as the main barrier to nicotine absorption [32]. This model holds promise for applications in designing transdermal delivery systems, particularly for estimating how heat affects systemic drug levels in experimental IVPT data and systemic PK data. The potential of AI in resolving the drug delivery issues in transdermal drug delivery is indicated in tabular column 5 (Table 5).

## 4. Future Potential of AI in Overcoming Obstacles in TDDS

Though the engagement of ML and leveraging AI can accelerate the development of safe and effective disease treatments, a few drawbacks are addressed in tabular column 6 (Table 6).

With advancements in AI, these drawbacks could be resolved. TDDS can become more efficient, precise, and adaptable, paving the way for breakthroughs in drug delivery technology. For instance, integrating AI with nanotechnology in TDDS may lead to novel delivery platforms that can target diseases with unparalleled specificity.

## Figures and Tables

**Table 1:** Transdermal drug delivery systems (TDDS) for common viral infectious diseases.

Cargo	Indication	TDDS	References
Acyclovir	HSV disease	Binary Ethosome TDDS	[39]
Acyclovir	HSV disease	Water-in-oil microemulsion	[44]
Acyclovir	HSV disease	gel plaster	[48]
Penciclovir	HSV disease	Hydrogel Nanoemulsion	[43]
Vaccine	Influenza Virus Disease	Egg microneedles	[45]
Zanamivir	Influenza Virus Disease	Transdermal patch-control release	[46]
5-FU	Warts’ disease	Solid microneedles	[40]
SARS-CoV-2 Vaccine	COVID-19 disease	Fluorocarbon-modified chitosan (FCS)based transdermal delivery	[47]
SARS-CoV-2 Vaccine	COVID-19 disease	Microneedles TDDS	[42]

**Table 2:** Transdermal drug delivery systems (TDDS) for Central Nervous System (CNS) disorders.

Drug	Indication	TDDS	References
Nicotine	Dementia, Alzheimer’s disease	Transdermal patch-matrix	[59]
Physostigmine	Alzheimer disease	Transdermal patch-control release	[60,61]
Phenserine	Cognition impairment	Transdermal patch-reservoir type	[62]
Rivastigmine	Behavioral disorders	Transdermal patch-control release	[63]
Tacrine	Alzheimer disease	TDDS by iontophoresis	[64]
Sumatriptan	Migraine	Transdermal patch-control release	[65]
Pramipexole	Parkinson’s disease	Transdermal patch	[66]
Donepezil	Alzheimer disease	Transdermal patch	[67]
Rotigotine	Parkinson’s disease	Transdermal patch	[68]
Everolimus	Multiple sclerosis	Microneedle-based transdermal patch	[69]
Baclofen	Multiple sclerosis	Nanosuspension-based microneedle skin patch	[70]
Asenapine	Schizophrenia	Transdermal patch	[71]
Blonaserin	Schizophrenia	Transdermal patch	[72]
Clozapine	Schizophrenia	Nanoemulsion-based transdermal drug delivery system	[73]
Risperidone	Antipsychotic	Transferosomal gel-transdermal	[74]
Olanzapine	Antipsychotic	Transferosomal TDDS	[75]
Quetiapine	Antipsychotic	Liposomal TDDS	[76]

**Table 3:** Transdermal drug delivery systems (TDDS) for Cardiovascular (CV) diseases.

Drug	Indication	TDDS	References
Nitroglycerin	Angina pectoris	Microneedle Transdermal patch	[85]
Propranolol	Antihypertensive	Gel-based transdermal patch	[98]
Bisoprolol	Atrial fibrillation	Gel-based transdermal patch	[99]
Clonidine	Attention-deficit hyperactivity disorder	Polymeric transdermal patch	[96]
Losartan	Antihypertensive	Proniosome TDDS	[97]
Aspirin	Antithrombotic	Hydrocarbon gel-based TDDS	[100]
Clopidogrel	Antithrombotic	Polymeric transdermal patch	[101]
Pravastatin	Antithrombotic	emulgel based transdermal patch	[102]
Simvastatin	Hypercholesterolemia	Nanostructured lipid carrier-based TDDS	[103]
Lisinopril	Congestive heart failure	Lipid-based transdermal gels	[104]
Atorvastatin	Hypercholesterolemia	Microneedle based TDDS	[105]
Verapamil hydrochoride and Amlodipine besylate	Chronic hypertension	Microneedle-based transdermal patch	[106]
Diltiazem and Nifedipin	Chronic hypertension	Nanosphere-based microneedle TDDS	[107]
Sodium nitroprusside	Hypertensive emergency	Dissolvable microneedle patch	[108]

**Table 4:** Transdermal drug delivery systems (TDDS) for hormonal imbalance and autoimmune disorders.

Drug	Indication	Formulation	References
Estrogen, Progesterone	Hormonal imbalance, Menopause	Transdermal patches, gels	[111,113,114]
Testosterone	Hormonal imbalance	Transdermal gels, patches	[115,116,117]
Hydrocortisone	Inflammation, Hormonal imbalance	Transdermal creams, gels	[118,119]
Methotrexate	Autoimmune Rheumatoid Arthritis (RA)	Transdermal patches	[119]
Hydrocortisone	Autoimmune (RA), inflammation	Transdermal patches, creams	[120]
Diclofenac (NSAID)	Autoimmune (RA) pain relief	Transdermal patches	[121]
Etanercept (TNF inhibitor)	Autoimmune (RA)	Transdermal patches, microneedles	[122,123]
Adalimumab (TNF inhibitor)	Autoimmune (RA)	microneedle	[124]

**Table 5:** Approaches to resolving problems associated with developing TDDS.

Problem	Solution	Approach	Datasets	Models	Results	References
Screening of drug molecules and optimizing formulation of skin delivery	Predict the aqueous solubility of drug compounds.	Develop and evaluate machine learning models using diverse data and representations (ESP maps, molecular graphs, and tabular features).	Four solubility datasets (ESOL, AQUA, PHYS, OCHEM) with 3942 unique molecules.	Graph Convolutional Network (GCN) for molecular graphs, EdgeConv for ESP maps XGBoost with selected tabular features Ensemble of all three models	Provides a valuable tool for drug discovery. Enables early identification and mitigation of solubility issues. Improves the efficiency of drug development processes	[143]
skin permeability is crucial for successful TDDS.	Improve accurate prediction of skin permeability	Evaluate the accuracy of machine learning models (LGBM, XGBoost, CatBoost) on skin permeability.	Compiled a dataset of 441 molecules with diverse skin permeability values (LogKp). Identified the key descriptors’ effects (hydrophobicity, hydrogen bonding, and polar surface area on skin permeability).	Employed various machine learning models (MLR, RF, XGBoost, CatBoost, LGBM, ANN) for regression analysis.	LGBM, XGBoost, CatBoost) showed superior predictive accuracy. Key descriptors like hydrophobicity, hydrogen bonding, and topological polar surface area significantly influence permeability.	[144]
*Simulation and Virtual Testing:*	Simulated values for key pharmacokinetic parameters. and Predicted drug concentrations.	Development of “BIOiSIM,” a physiologically based transdermal platform for predicting drug exposure	BIOiSIM model utilizes human clinical trials, including drug concentration-time profiles (plasma, tissues), preclinical data, and data from animal studies, including pharmacokinetic parameters and pharmacodynamic responses.	BIOiSIM platform includes: 16-compartment model, a structure representing the pharmacokinetic processes within the body, and Machine learning algorithms.	Accurate Predictions: The model demonstrated good accuracy in predicting drug exposure (AUC, Cmax, Tmax) for three different drugs (morphine, buprenorphine, and nicotine) compared to clinical data.	[140]

**Table 6:** Pros and Cons of AI in TDDS.

Pros	Cons
Enhanced Formulation Accuracy: AI models can analyze vast datasets of formulation parameters (e.g., drug properties, excipients, manufacturing conditions) and predict optimal combinations [145]. Identify complex interactions between formulation components that may not be apparent to human experts [144]. Improved Success Rate: AI models can predict the likelihood of successful TDD formulations based on historical data and identify potential pitfalls. Help minimize formulation failures and reduce the risk of expensive clinical trials. Reduced Experimental Costs: AI can significantly reduce research and development costs by accelerating the formulation development process and minimizing the number of costly experiments [30].	Data Dependence: Relies heavily on high-quality, diverse, and unbiased training data. Limited or biased data can lead to inaccurate predictions [145]. Model Complexity: Some AI models, particularly deep learning models, can be complex and difficult to interpret. Limited Generalizability: Models may overfit the training data, leading to poor performance on new, unseen formulations. e.g., Overfitting in Deep Neural Networks (DNNs) [146]. Computational Cost: Concerns about data privacy and security when using patient data to train AI models. Data sensitivity: Some AI tools are noise Sensitivity, which means errors or random fluctuations can negatively impact AI performance. Noisy data can lead to overfitting, where the model performs well on the training data but poorly on new, unseen data. e.g., data sensitivity in SVMs. [147,148]
